# Intussusception induced by gastrointestinal metastasis of malignant melanoma: A case report

**DOI:** 10.1016/j.ijscr.2020.03.026

**Published:** 2020-04-01

**Authors:** Koichiro Kumano, Tsuyoshi Enomoto, Daichi Kitaguchi, Yohei Owada, Yusuke Ohara, Tatsuya Oda

**Affiliations:** University of Tsukuba, Faculty of Medicine, Gastroenterological and Hepato-Biliary-Pancreatic Surgery, Tennnodai, Tsukuba-city, Ibaraki-Ken, 305-8575, Japan

**Keywords:** Malignant melanoma, Intussusception, Metastatic small bowel tumor, Case report

## Abstract

**Introduction:**

Malignant melanoma sometimes metastasizes to small intestine, and could cause various clinical symptoms, including intussusception. Among the acute abdomen cohort in Japan, it is quite rare to encounter this entity.

**Presentation of case:**

A 68-year-old male patient was admitted to our hospital with chief complaints of abdominal pain and vomiting.　He underwent tumor resection for malignant melanoma of the primary lesion at left foot base, local recurrence and brain metastasis during the last five years. At admission, abdominal X-ray demonstrated small bowel obstruction. An ileus tube was inserted, and contrast media enema study showed crab-like shadow defect was observed in the advanced part. Enhanced computed tomography showed intussusception in the proximal jejunum caused by a tumor of 5 cm in diameter in the advanced part. No other intestinal lesion was found. Diagnosis of intussusception caused by solitary metastasis of malignant melanoma was made. Laparoscopic partial resection of the small intestine was performed. Postoperative course was uneventful, and patient was followed in outpatient clinic without further treatment with any recurrence of disease for one years.

**Discussion:**

Malignant melanoma tends to metastases to the small intestine simultaneously and multiply. It bothers surgeons to decide range of small intestinal resection at emergency surgery. In the present study, preoperative examination allowed the adequate range of intestinal resection including location and number of metastases before operation.

**Conclusion:**

When an acute abdomen caused by intestinal metastasis of malignant melanoma was consulted, surgeon should make effort to identify location and number of metastatic lesion, preoperatively.

## Highlights

• Diagnosis of intestinal metastasis of malignant melanoma is difficult because it usually made after appearing symptoms of bowel obstruction.
• Emergency surgery for malignant melanoma of small intestine are often performed without enough examination preoperatively.
• Malignant melanoma tends to metastases to the small intestine simultaneously and multiply.
• When treating small intestinal metastasis of malignant melanoma, location and number of the metastasis should be understood.

## 1 Introduction

Malignant melanoma is a rare disease derived from melanocytes that occurs in parts of the body where melanocytes are present, such as the skin, eye socket, oral cavity, nasal mucosa [1].

Malignant melanoma is characterized by its high metastatic ability and a poor prognosis with a 5-year survival rate of only 9–13% [1]. Malignant melanoma rapidly spreads to many organs and gastrointestinal metastasis is recognized in approximately 60% of autopsied Malignant melanoma patients [2].

Bowel obstruction is a representative symptom of intestinal metastasis of Malignant melanoma and intussusception is most frequent cause in this entity [3]. Emergency operation are needed, however, diagnosis of intestinal metastasis of malignant melanoma is difficult preoperatively. Operation is often performed without enough examination, as a result in difficulty of determination of the range of resection, especially in the case of multiple metastatic lesions.

We experienced a case of metastatic malignant melanoma of the small bowel with intussusception. We herein report our experience with the present case with consideration of the relevant literature. This work has been written in accordance with the SCARE criteria [4].

## 2 Presentation of case

A 68-year-old man was admitted to our department with chief complaints of abdominal pain, and vomiting. Five years previously, he had undergone resection of malignant melanoma of the left foot base with dissection of inguinal, popliteal, pelvic lymph nodes. The postoperative pathological stage was cT4bN2M0 according to the UICC classification. Five courses of chemotherapy followed by interferon treatment were administered for 2 years after the operation.

Relapse occurred at the left inguinal region and external left lateralis muscle at three years after the first operation. The second operation was performed, and metastatic lesion was completely resected, however, at three months after second operation, brain metastasis recognized with symptoms of right hemiplegia. The third operation of resection of metastatic brain tumor was performed.

At two years after the third operation, the patient visited the emergency department of our hospital with abdominal pain and vomiting. At the time of admission, a physical examination showed marked abdominal distension without tenderness. In the left lower quadrant and right axilla region, a metastatic tumor of malignant melanoma with 2 cm in diameter were also palpated. A blood test showed mild anemia with a serum hemoglobin concentration of 11 g/dl. Abdominal X-ray demonstrated dilatation of the small intestine; based on these findings, a diagnosis of ileus was made.

**Fig. 1:**
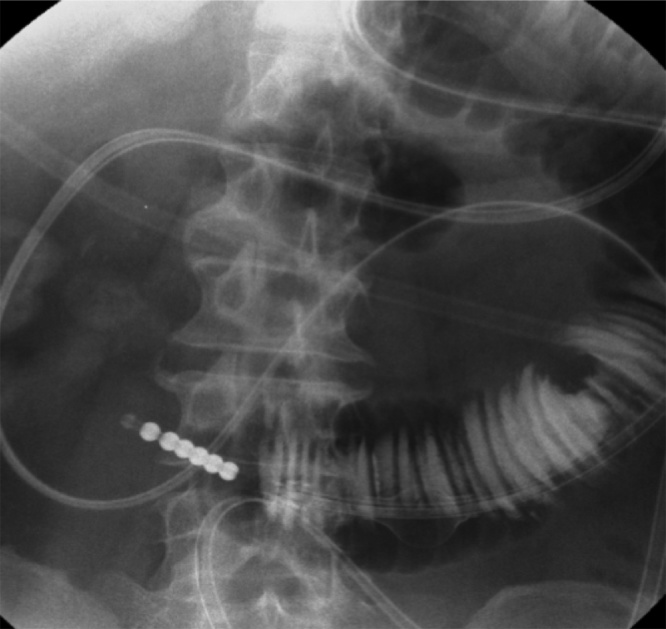
The enema study revealed complete obstruction in the advanced part of the ileus tube at the proximal portion to the jejunum.

**Fig. 2:**
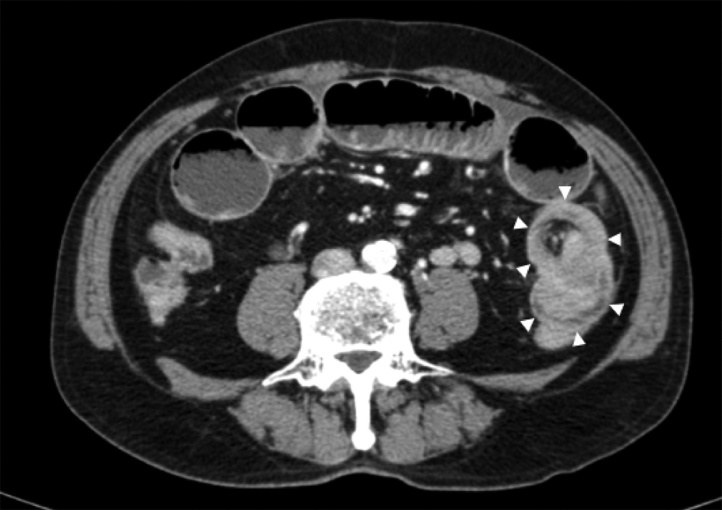
Enhanced abdominal computed tomography detected intussusception in the left lower quadrant with a tumor of 5 cm in diameter in the advanced part (white arrow head).

An ileus tube was inserted into the small intestine to decompress the intestinal contents, and a contrast media enema (Gastrographin) study was performed to clarify the exact location of the obstruction. The enema study revealed complete obstruction in the advanced part of the ileus tube at the proximal portion of the jejunum (Fig. 1).　Enhanced abdominal computed tomography detected intussusception in the left lower quadrant with a tumor of 5 cm in diameter in the advanced part. The oral side of small intestine showed marked dilatation (Fig. 2).

Based on the above findings, a diagnosis of intussusception due to metastatic malignant melanoma of the small intestine was made.

**Fig. 3:**
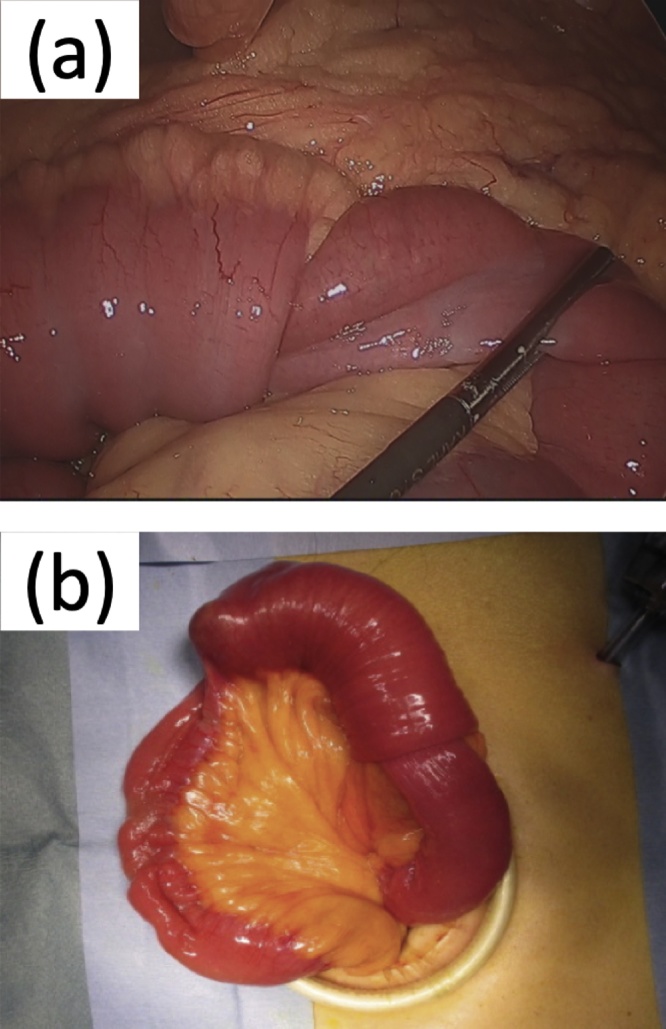
(a), (b): Laparoscopy shows intussusception at the jejunum, 60 cm from the Treitz ligament.

After ileus tube insertion, abdominal distension was markedly improved, and the contents of the small intestine were almost completely drained; therefore, laparoscopic partial resection of the small intestine was performed. A small incision was made at the umbilicus, and pneumoperitoneum was induced via the direct peritoneal access (Hasson) technique. Two additional 5-mm trocar ports were inserted (1 each at the upper and lower right abdomen). The surgical findings revealed intussusception 60 cm from the start of the jejunum (Fig. 3a and b). Intussusception was difficult to reduce manually, jejunal resection was performed without the release of the intestinal tract. Partial resection of the small intestine, including intussusception and handsewn anastomosis, was performed extracorporeally.

**Fig. 4:**
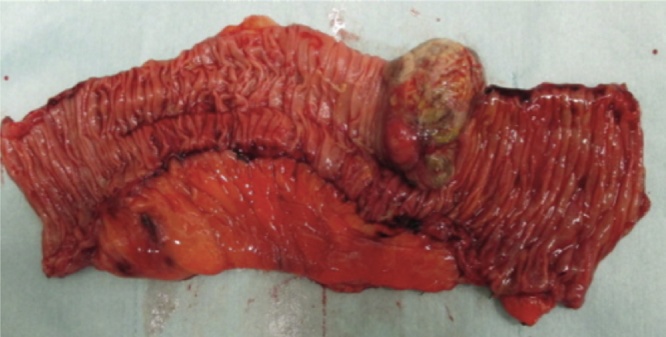
The tumor is 5 cm × 3 cm in size. The tumor has a blackish appearance and its surface is necrotic.

The resected specimen showed a round metastatic tumor of 5 × 3 cm in size, with a blackish appearance and necrotic tissue on the surface (Fig. 4).

**Fig. 5:**
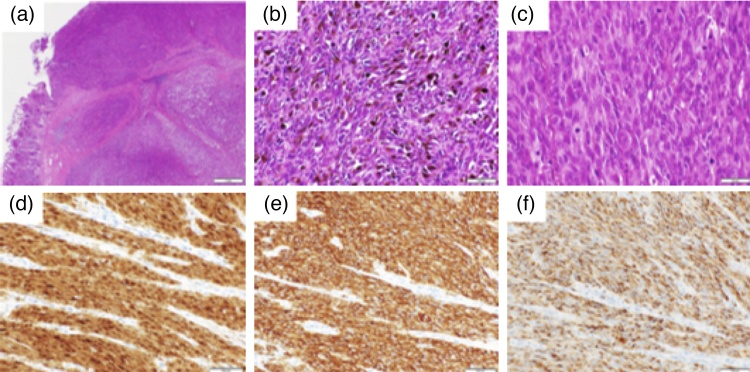
(a)–(c) Large and small asymmetric tumors with distinct nucleoli and large tumor cells with melanin deposition were observed. Immunohistochemical staining reveals that the tumor is (d) S-100 (+), (e) HMB-45 (+), (f) Melan-A (+).

A histopathological examination revealed large and small asymmetric tumors with distinct nucleoli and large tumor cells with melanin deposition. Immunohistochemical was positive for S-100, HMB-45, and Melan-A. Based on these findings, the tumor was diagnosed as small intestinal metastasis of malignant melanoma (Fig. 5a–e). The postoperative course was good, and the patient was discharged on the 13th postoperative day. The patient was followed in an outpatient clinic without further treatment with any recurrence of disease for one years.

## 3 Discussion

The most frequent site of gastrointestinal metastasis of malignant melanoma is the small intestine (71–91%), followed by the colon (22–26%) [[5], [6], [7]]. Hematogenous metastasis is the main metastatic form of malignant melanoma; thus, metastasis is most likely to occur in the small intestine where the blood flow is abundant [8].

The frequency of gastrointestinal metastasis is recognized in 60% of autopsied malignant melanoma cases [9]. On the contrary symptoms of abdominal pain, vomiting and bleeding rarely occur during the clinical course and only 0.8–9% of cases are diagnosed preoperatively [2].

The diagnosis of malignant melanoma of the small intestine largely depends on the findings of enhanced CT. An enema study has an advantage in that it can identify the location of obstruction in the small intestine before surgery. A contrast examination can more precisely pinpoint the site of a tumor than CT [10].　Recent advances in diagnostic modalities, including FDG-PET, capsule endoscopy, and double-balloon endoscopy have facilitated the early diagnosis of intussusception, however, indication of these modalities is quite limited in clinical course.

The treatment for small bowel malignant melanoma is surgical resection or chemotherapy, surgical resection is the main treatment for this disease. The postoperative prognosis of gastrointestinal metastasis of malignant melanoma is quite poor, with a median survival of 6.2–13 months [8,11]. When complete resection is performed, the median survival time is improved as 14.9–44.5 months and the 5-year survival rate is 28.3–35% (some cases have remained alive for 25 years) [8,11]. On the contrary, the possibility has been proposed that surgical invasion may lead to multiple metastases in the early period after surgery, which can lead to a reduced survival time [12]. Elsayed reported that younger age and early metastasis from the first treatment are poor prognostic factors [13]. Chemotherapy, DAV-Feron, CDV (Cisplatin, Dacarbazine, Vincristinesulfate), DAC-Tam (Dacarbazine, Nimustine hydrochloride, Cisplatin-Tamoxifen) therapy are usually administered in the clinical setting; however, the response rate is 10–30% and it has limited effect on distant metastasis [14,17,15].

Between 2008–2019, 36 cases (including our own) were identified in a search of the PubMed database using the keywords “malignant melanoma and intussusception”. All cases required surgical treatment. The male/female ratio was 1:1, and the average age was 53 years (range: 20–85 years). Twenty-seven of the patients had metastatic lesions and the average periods from the first treatment to operation was 80 months. Half of the cases had multiple lesions in the intestinal tract and many cases were first found during surgery. Almost all patients received open surgery; four patients underwent laparoscopic surgery. The value of laparoscopic surgery in emergency or reoperation has been reported [16]. In the present study, decompressing the intestinal contents before surgery allowed us to identify the location of the lesion, determine which part of the intestine to resect and operate with minimal invasiveness by laparoscopic surgery.

## 4 Conclusion

A case of intussusception induced by small bowel metastasis of malignant melanoma was reported. Malignant melanoma tends to metastases to the small intestine simultaneously and multiply, therefore when treating intussusception induced by small intestinal metastasis of malignant melanoma, location and number of the metastasis should be understood before surgery because it is too difficult to decide adequate number and range of intestinal resection intraoperatively. In this case, the ileus tube was inserted prior to the operation, and the number and location of lesion was firmly identified. Surgery was performed after confirming the necessary range of intestinal resection in advance.

## Sources of funding

Source of funding is none.

## Ethical approval

This report is not research study.

## Consent

Written informed consent was obtained from the patient for publication of this case report and accompanying images. A copy of the written consent is available for review by the Editor in Chief of this journal on request.

## Author contribution

Kumano carried out revision of the manuscript. Tsuyoshi Enomoto is the corresponding author and All authors read, supervised the writing of the manuscript and approved the final manuscript.

## Registration of research studies

This report is not research study.

## Guarantor

Tsuyoshi ENOMOTO.

## Provenance and peer review

Not commissioned, externally peer-reviewed.

## Declaration of Competing Interest

There is no conflict of interest.
